# Metastable CrMnNi steels processed by laser powder bed fusion: experimental assessment of elementary mechanisms contributing to microstructure, properties and residual stress

**DOI:** 10.1038/s41598-022-26052-x

**Published:** 2022-12-18

**Authors:** J. Richter, G. Bartzsch, S. Scherbring, A. Bolender, M. Vollmer, J. Mola, O. Volkova, T. Niendorf

**Affiliations:** 1 Institute of Materials Engineering – Metallic Materials, University of Kassel, Moenchebergstrasse 3, 34125 Kassel, Germany; 2 Institute of Iron and Steel Technology, TU Bergakademie Freiberg, Leipziger Strasse 34, 09599 Freiberg/Saxony, Germany; 3 Faculty of Engineering and Computer Sciences, Materials Design and Structural Integrity Laboratory, Osnabrueck University of Applied Sciences, Albrechtstrasse 30, 49076 Osnabrueck, Germany

**Keywords:** Mechanical properties, Metals and alloys, Mechanical engineering

## Abstract

The complex thermal history imposed by the laser-based powder bed fusion of metals (PBF-LB/M) process is known to promote the evolution of unique microstructures. In the present study, metastable CrMnNi steels with different nickel contents and, thus, different phase stabilities are manufactured by PBF-LB/M. Results clearly reveal that an adequate choice of materials will allow to tailor mechanical properties as well as residual stress states in the as-built material to eventually redundantize any thermal post-treatment. The chemical differences lead to different phase constitutions in as-built conditions and, thus, affect microstructure evolution and elementary deformation mechanisms upon deformation, i.e., twinning and martensitic transformation. Such alloys designed for additive manufacturing (AM) highlight the possibility to tackle well-known challenges in AM such as limited damage tolerance, porosity and detrimental residual stress states without conducting any post treatments, e.g., stress relieve and hot isostatic pressing. From the perspective of robust design of AM components, indeed it seems to be a very effective approach to adapt the material to the process characteristics of AM.

## Introduction

Within the last decade additive manufacturing (AM) processes, such as laser-based powder bed fusion of metals (PBF-LB/M) (also known as laser powder bed fusion (LPBF) or selective laser melting (SLM)), evolved from techniques used only for prototyping to direct manufacturing. The layer-wise built-up combined with the unprecedented freedom of design are appealing for many industries, e.g., aerospace and medical engineering. Especially tool-free manufacturing is an advantage, e.g., in terms of individualization, small batch productions and advanced topology optimization being of highest importance for light-weight parts^1^.

Process-inherent cooling conditions and melt pool dynamics, respectively, in the PBF-LB/M process often result in unique microstructures; however, advantageous features are usually accompanied by detrimental residual stresses and material defects like porosity^2–4^. The high stresses can be attributed to the small melt pool sizes and high cooling rates. Porosity often stems from inappropriate process parameter combinations or can be introduced by the powder itself. Driven by different industrial sectors, titanium alloy Ti6Al4V, nickel-based superalloy Inconel 718 (IN718) and austenitic stainless steel 316L were in the focus of numerous studies and relevant process-property-relationships were studied in detail^5–8^. Within the last years, the range of alloys processed by AM technologies has been extended rapidly, e.g., aluminum alloys, tool steels and even smart materials came into focus of research^9–13^. AM metals can be characterized by mechanical properties being different from conventionally manufactured counterparts, e.g., increased strength or even a change in Young’s modulus^14,15^. In the PBF-LB/M process, the as-built microstructure of 316L-type alloys tends to develop coarse grains. These grains, which are mainly elongated in build direction (BD), lead to the development of a preferred crystallographic orientation, eventually resulting in anisotropic mechanical properties^16,17^. The development of such anisotropic microstructures is mainly attributed to the directional heat flow, epitaxial solidification/growth and rapid cooling as well as the absence of any phase transformation within the cooling process. Similar microstructural evolution, i.e., coarse grains and strong texture, were shown for IN718 processed by PBF-LB/M and electron-beam powder bed fusion of metals (PBF-EB/M) as well^18,19^. Austenitic steels with coarse grains usually show a high ductility at the expense of strength. However, additively manufactured 316L shows significantly higher yield strength (YS) combined with high ductility when compared to conventionally manufactured counterparts. This is attributed to subgrain structures eventually increasing the strength in accordance to the Hall–Petch relation^20^. Therefore, PBF/LB-M represents a promising process to overcome the strength-ductility trade-off^21,22^.

In contrast to face-centered cubic (fcc) austenitic steels, body-centered (bcc) pure iron and tool steels are characterized by a relatively fine-grained microstructure upon AM being attributed to the occurrence of multiple phase transformations imposed by intrinsic heat treatment and cooling^23–28^. Similar to those bcc steels, recurring phase transformations induced by intrinsic heat treatment (being characteristic for all AM processes) were also reported by Guenther et al.^29^ in an austenitic CrMnNi steel processed by PBF-EB/M, eventually resulting in an isotropic, equiaxed, fine-grained microstructure. The evolution of the equiaxed fine structure was rationalized by repeated fcc ↔ fcc + bcc phase transformations occurring within the process at relatively high temperatures^30^.

Under monotonic loading, CrMnNi steels exhibit a high defect tolerance due to the transformation induced plasticity effect (TRIP) being characteristic for such kind of metastable austenitic steels^29^. The outstanding damage tolerance is beneficial to balance process induced defects under monotonic loading as well as fatigue loading, wherefore the material is highly suitable for AM^31,32^. In general, the chemical composition highly influences microstructure and mechanical properties of an alloy due to a direct influence on the stacking fault energy (SFE)^33,34^. Generally, the SFE strongly affects the dominant deformation mechanism in the material. Deformation is accommodated by the motion of perfect dislocations in case of a high SFE. With a decrease of SFE, twinning and martensite formation come into play^35,36^. At room temperature (RT) merely perfect dislocation movement occurs above a SFE of 40 mJm^−2^, whereas phase transformation takes place below 20 mJm^−2^, eventually promoted by dissociated partial dislocations^37^. The simultaneous change of SFE and austenite stability, and the different contributions of the aforementioned elementary deformation mechanisms during loading at RT were already thoroughly studied for conventionally manufactured CrMnNi steel with 16 wt.%Cr, 6 wt.% Mn and varying nickel contents of 3, 6 and 9 wt.%. It was shown that the martensite start temperature drops with an increasing nickel content from 60 to − 47 °C^38^. Therefore, the microstructure after casting differs among these steels. The 9 wt.% composition is fully austenitic. After reduction of the nickel content to 6 wt.%, about 3 vol.% of δ-ferrite are present in the as-cast condition. The 3 wt.% nickel composition is characterized by 16 vol.% of δ-ferrite and even martensite is present in the as-cast condition^39^. The steel with 9 wt.% nickel is characterized by a SFE of 22 mJm^−2^. Thus, deformation is dominated by dislocation slip and twinning induced plasticity (TWIP) at RT. At a reduced nickel content of 6 wt.%, the formation of ε- and α′-martensite and twinning during deformation are strongly promoted. At 3 wt.% nickel, deformation leads to extensive ε- and α′-martensite formation. For the 6 wt.% and 3 wt.% nickel systems, the SFE was calculated with 16 and 10 mJm^−2^, respectively, at RT^40^.

Due to those different initial phases and deformation mechanisms, the ultimate tensile strength (UTS) and elongation at fracture are differing in the as-cast condition. The CrMnNi steel with 9 wt.% nickel shows the lowest strength and the highest ductility (550 MPa and 72%, respectively). The UTS for the steels with 6 wt.% and 3 wt.% nickel content are 765 MPa and 1013 MPa, respectively, at RT, whereas the elongations at fracture are 53% and 23%^41^. However, due to the initial coarse-grained as-cast microstructures the YS is relatively low (< 300 MPa) for all three chemical compositions^39,41,42^. Though the prevailing cast microstructure is coarse-grained, the combination of active deformation mechanisms leads to improvement of the mechanical properties (i.e. a dynamic Hall–Petch effect) resulting in a combination of high ductility and strength. The initial microstructure is intrinsically refined by the formation of deformation bands, strain induced martensite and mechanical twins, all features acting as obstacles to dislocation movement. Thus, pronounced strain hardening takes place.

As detailed, metastable CrMnNi steels were already extensively studied in conventionally manufactured conditions, eventually showing excellent ductility and strength directly tailorable by the variation of the nickel content. In direct comparison to CrNi steels, the advantage of an increased manganese content is the reduction of the expensive element nickel, in order to reduce costs, while enhancing the mechanical properties. However, in Am it it adventageous to replace the nickel only partially due to the high volatility of manganese^30^. The conventionally processed conditions suffer from a low YS, at least in the as-cast condition. By using PBF-EB/M, it was shown that a CrMnNi steel with 6 wt.% nickel forms an isotropic, equiaxed fine-grained microstructure. This was attributed to the process inherent thermal history, eventually leading to an increased YS^29^. In the present study, three 16Cr6MnXNi steels with X = 3, 6 or 9 (referred to as 16-6-3, 16-6-6 and 16-6-9 in the following) are manufactured via PBF-LB/M to investigate the processability of these highly promising representatives of TWIP/TRIP steels and to exploit the process-inherent characteristics, i.e. intrinsic heat treatment and rapid cooling, to finally establish microstructures being characterized by increased strength and high damage tolerance to eventually overcome the strength-ductility trade-off. The materials are characterized with focus on their microstructure, residual stress state and quasi-static mechanical properties to evaluate the influence of different phase stabilities. The results are compared to those of conventionally manufactured specimens from literature.

## Results

The chemical compositions of initial powders and PBF-LB/M bulk materials given in Table 1 were analyzed with respect to chromium, manganese and nickel as well as carbon and nitrogen for the bulk materials. All three bulk compositions are characterized by a low amount of nitrogen and carbon. For the material with 3 wt.% Ni, the carbon is slightly increased compared to the other materials. Focusing on the main alloying elements, the overall chemical composition of the powders deviates from the bulk materials. Whereas the chromium and nickel content of all PBF-LB/M materials are close to the intended chemical compositions in both powder and bulk material, manganese is depleted during PBF-LB/M processing.

**Table 1:** Chemical composition of the initial powders and the PBF-LB/M manufactured parts given in wt.%.

		C	N	Cr	Mn	Ni	Fe
16-6-9	Powder			15.96	5.95	9.01	Bal
	PBF-LB/M	0.05	0.04	15.68	4.95	9.12	Bal
16-6-6	Powder			15.96	6.29	6.14	Bal
	PBF-LB/M	0.05	0.04	16.08	5.82	6.18	Bal
16-6-3	Powder			16.50	6.12	3.42	Bal
	PBF-LB/M	0.08	0.04	16.21	5.49	3.37	Bal

For the PBF-LB/M bulk materials also carbon and nitrogen were analyzed due to their potential influence on the finally prevailing SFE (being of highest importance for the properties of the conditions studied). For the powder materials the carbon and nitrogen contents were not analyzed.

**Figure 1:**
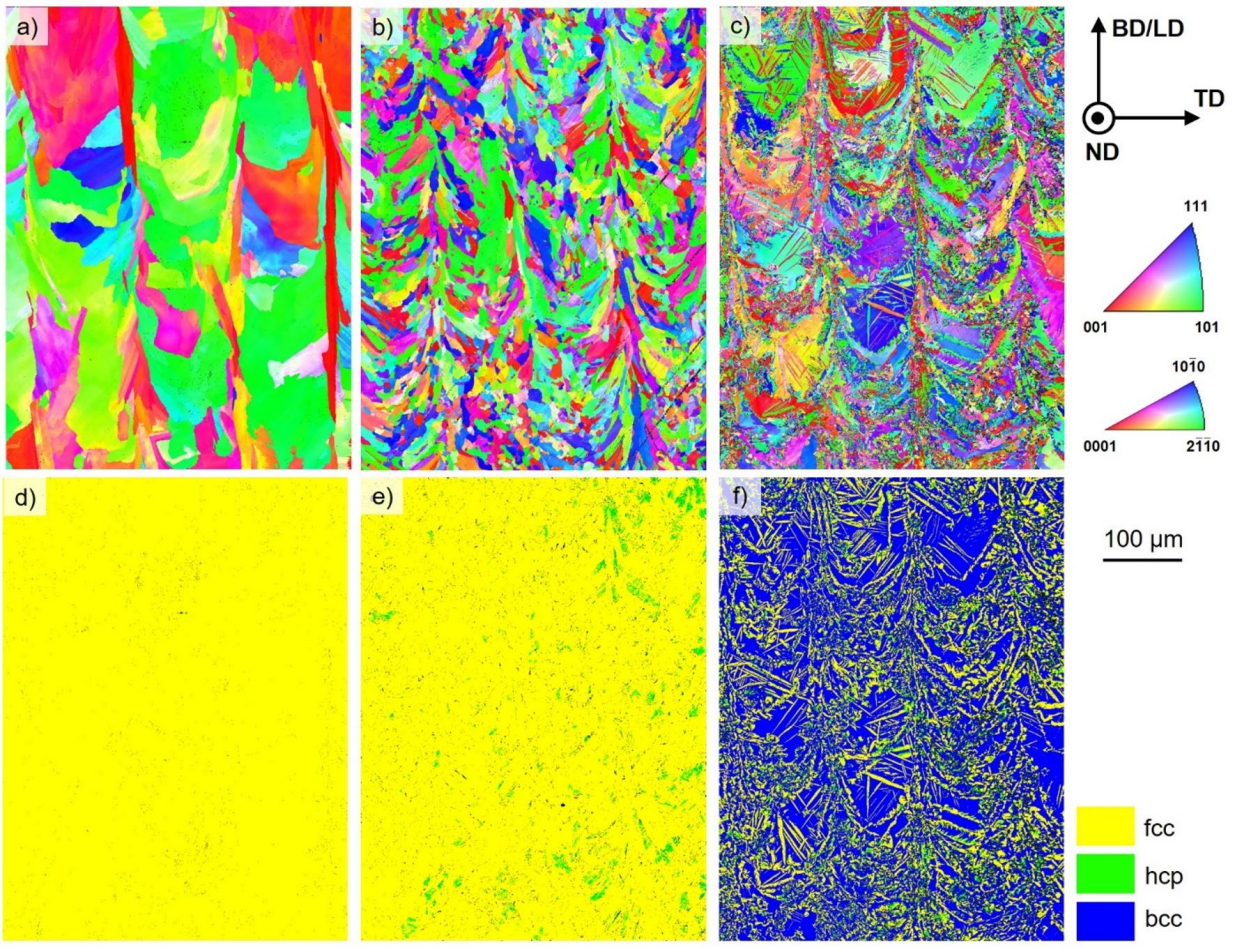
EBSD orientation maps plotted with respect to build direction || loading direction (BD || LD) and corresponding phase maps of PBF-LB/M-16-6-9 (**a**, **d**), PBF-LB/M-16-6-6 (**b**, **e**), PBF-LB/M-16-6-3 (**c**, **f**). In dependence of the alloy composition face-centered cubic (fcc), body-center cubic (bcc) as well as hexagonal closed-packed (hcp) phases are seen. The average grain sizes of the 16-6-9 and 16-6-6 alloys are 73.1 µm ± 22.6 µm and 15.8 µm ± 5.1 µm, respectively. The average grain size for the bcc phase is about 21.8 ± 3.4 µm in the 16-6-3 composition, whereas the fcc and hcp phases being present have a considerably smaller grain size with 6.3 µm ± 1.7 µm and 2.7 µm ± 0.7 µm, respectively. The step size for all maps was 0.5 µm.

The grain orientation maps plotted with respect to BD obtained by electron backscattered diffraction (EBSD) are given in Fig. 1. The steel with the highest nickel content (9 wt.%) is characterized by a coarse-grained microstructure (Fig. 1a). Individual grains exceed the size of several hundred microns with a width of about 100 µm. The steel with 6 wt.% nickel content, shown in Fig. 1b, is characterized by a different microstructure with refined, randomly textured grains (cf. Supplementary data Fig. S2). Still, prevailing grains are slightly aligned following the shape of the preceding individual melt pools (these being indirectly visible in the map). For the additively manufactured 16-6-3 shown in Fig. 1c, the microstructure is composed of two different characteristic features. On the one hand, U-shaped coarse grains without pronounced texture are formed (pinpointing a melt pool width of about 100 µm). On the other hand, fine grains and needles, respectively, occur with different sizes. These features are either bundled alongside the preceding melt pool boundaries or intersecting the U-shaped grains.

In addition to the orientation maps, phase maps obtained by EBSD are depicted (Fig. 1d–f). With respect to accuracy and resolution of EBSD data, it is found that the steel with 9 wt.% Ni seems to be fully austenitic (Fig. 1d), whereas the PBF-LB/M-16-6-6 material shows a fcc microstructure with a small amount of ε-martensite (being indexed as hexagonal closed-packed (hcp) phase) in Fig. 1e. In contrast, the major fraction of the PBF-LB/M-16-6-3 material is bcc. Here, the bcc matrix is intermingled with fcc and hcp phases as shown in Fig. 1f.

**Figure 2:**
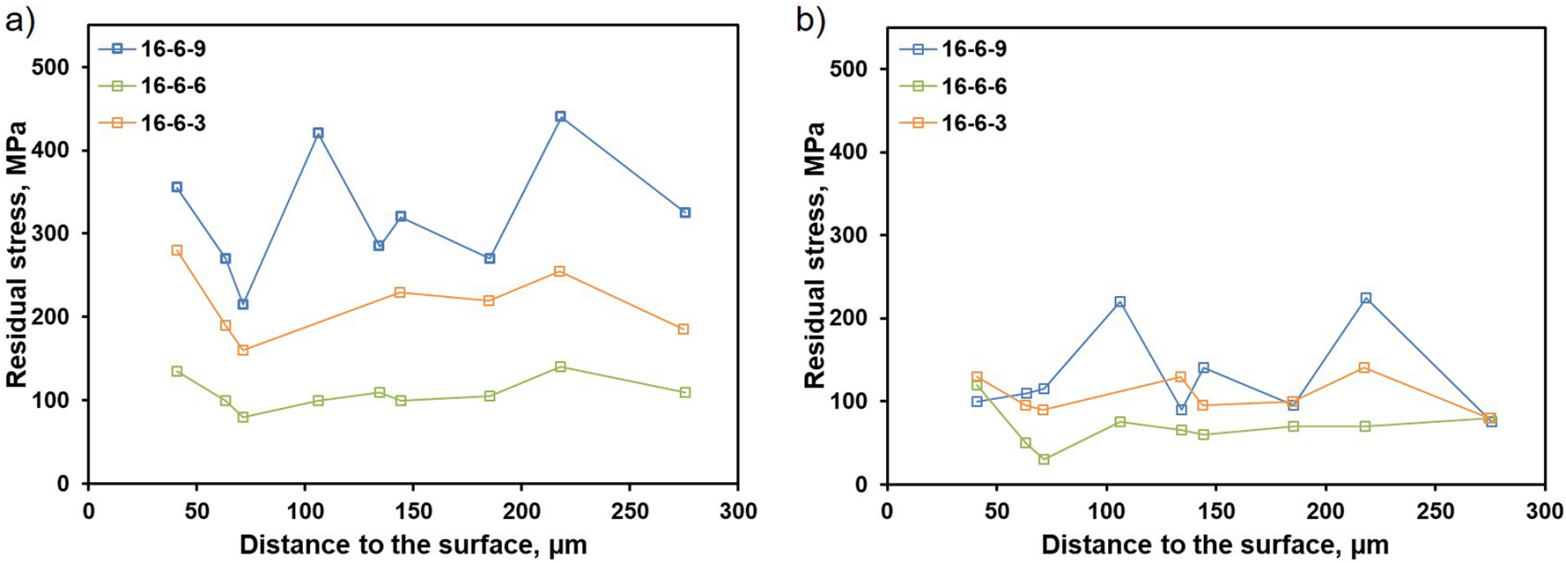
Near surface residual stress distribution determined as a function of distance to the surface by ED-XRD analysis for PBF-LB/M material of all three steels with respect to BD (**a**) as well as in TD (**b**).

The residual stress depth profiles, shown in Fig. 2, were determined by energy dispersive synchrotron diffraction (ED-XRD) in reflection mode in accordance to^43,44^. Data are plotted for the fcc phase. The stresses in BD and in transversal direction (TD) are shown in Fig. 2a and b, respectively. The stresses in normal direction are considered to be zero due to the plane-stress condition prevailing at free surfaces. In BD, the different alloys show highly diverging tensile stresses. In TD, the stresses reveal only small differences. The 9 wt.% Ni steel shows the highest residual stresses in BD and TD. The high scattering of stress values between 210 and 440 MPa can be attributed to coarse grains^45^. The material with 6 wt.% nickel shows the lowest absolute residual stresses of about 100 MPa in BD as well as TD. The PBF-LB/M 16-6-3 is characterized by intermediate residual stress values. The latter conditions are characterized by a less pronounced scattering.

Since the microstructure of the 16-6-3 steel is mainly ferritic, an assessment of residual stresses was additionally accomplished for the bcc phase (cf. Supplementary data, Fig. S3)^46^ as ED-XRD allows to differentiate between type I and type II residual stresses. Type I stresses are averaged across several grains and, thus, of highest importance in terms of numerous applications due to their long-range character. Type II stresses can be different in individual grains and phases, respectively^47^, e.g. shown for multi-phase steels and rationalized by different coefficients of thermal expansion (CTE) of prevailing phases^48,49^. Results are not shown, however, clearly revealed that values determined in fcc and bcc phases are similar in the volume probed, i.e., no deviation due to phase specific residual stress could be derived in this condition.

**Figure 3:**
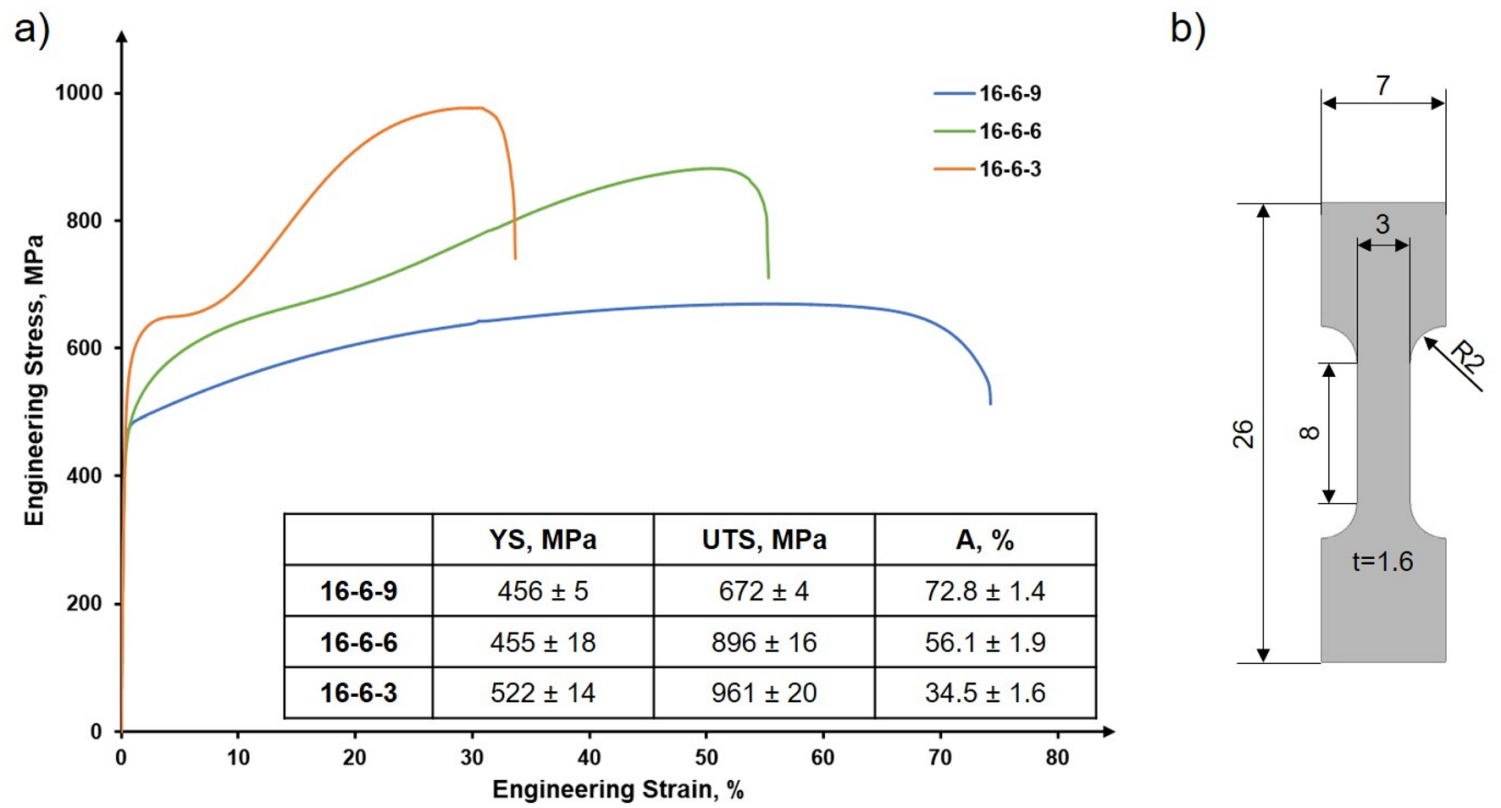
Stress-strain curves of PBF-LB/M-16-6-9, PBF-LB/M-16-6-6 and PBF-LB/M-16-6-3 (**a**); specimen geometry used for tensile tests (**b**). All dimensions are given in mm. The inset shows the average values as well as the standard deviations.

The deformation behavior under tensile loading is shown in Fig. 3a with the corresponding specimen geometry shown in 3b. Due to excellent repeatability, only one tensile test for each condition is shown for the sake of clarity. The lowest YS and UTS are seen for the PBF-LB/M-16-6-9 condition with 460 MPa and 670 MPa, respectively. Here, the strain at failure is more than 70%. The PBF-LB/M-16-6-6 condition shows a quite similar YS, but the UTS is increased up to 880 MPa. This specimen failed at a strain of 55%. The condition with 3 wt.% Ni is characterized by a considerably higher YS of 540 MPa, followed by a stress plateau at about 650 MPa up to a strain value of about 10%. Thereafter, pronounced strain hardening is seen leading to an UTS of 980 MPa, while the ductility at fracture is considerably lower with 33%. The true work hardening rate of all conditions can be found in the Supplementary data, Fig. S4.

**Figure 4:**
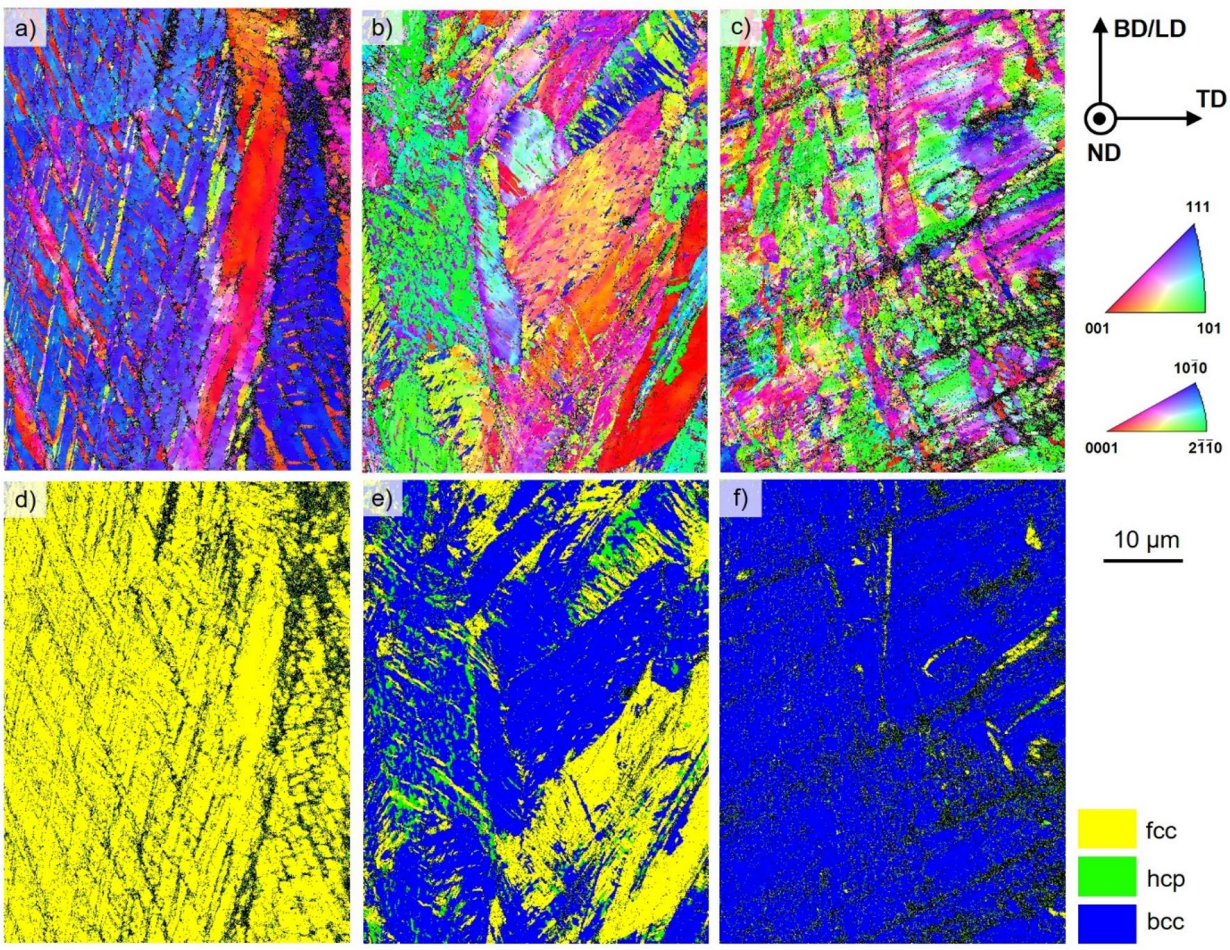
EBSD orientation maps of PBF-LB/M-16-6-9 (**a**), PBF-LB/M-16-6-6 (**b**), PBF-LB/M-16-6-3 (**c**) after tensile testing plotted with respect to BD || LD; the corresponding phase maps after deformation are shown in (**d**–**f**). Non-indexed areas revealed by black color are due to high local deformation. The step size for all maps was 0.075 µm.

The EBSD orientation maps of the failed specimens and the corresponding phase maps are shown in Fig. 4. It can be directly deduced from the phase map in Fig. 4d that the PBF-LB/M-16-6-9 steel remains fully austenitic after tensile testing. In the probed area, near-⟨111⟩-oriented grains seem to be intersected by near-⟨001⟩-oriented, acicular structures (cf. Fig. 4a). Moreover, needle-like <001>-structures can be found. Both structures represent most likely twins due to the orientation relationship determined (cf. Supplementary data, Fig. S5). The EBSD and phase maps for the steel 16-6-6 are given in Fig. 4b and e, respectively. Large grains, similar in size with respect to the initial microstructure, are visible. However, small grains with a size less than 1 µm inside those grains are also present. The microstructure mainly consists of bcc and fcc phases. Moreover, a small fraction of hcp phase is seen. In case of the 16-6-3 bulk material, the grains are highly fragmented and, therefore, considerably smaller in the examined area. Within each grain, many orientation deviations are obvious (cf. Fig. 4c). The dominant phase fraction is bcc with a minimum amount of fcc phase (cf. Fig. 4f).

## Discussion

**Figure 5:**
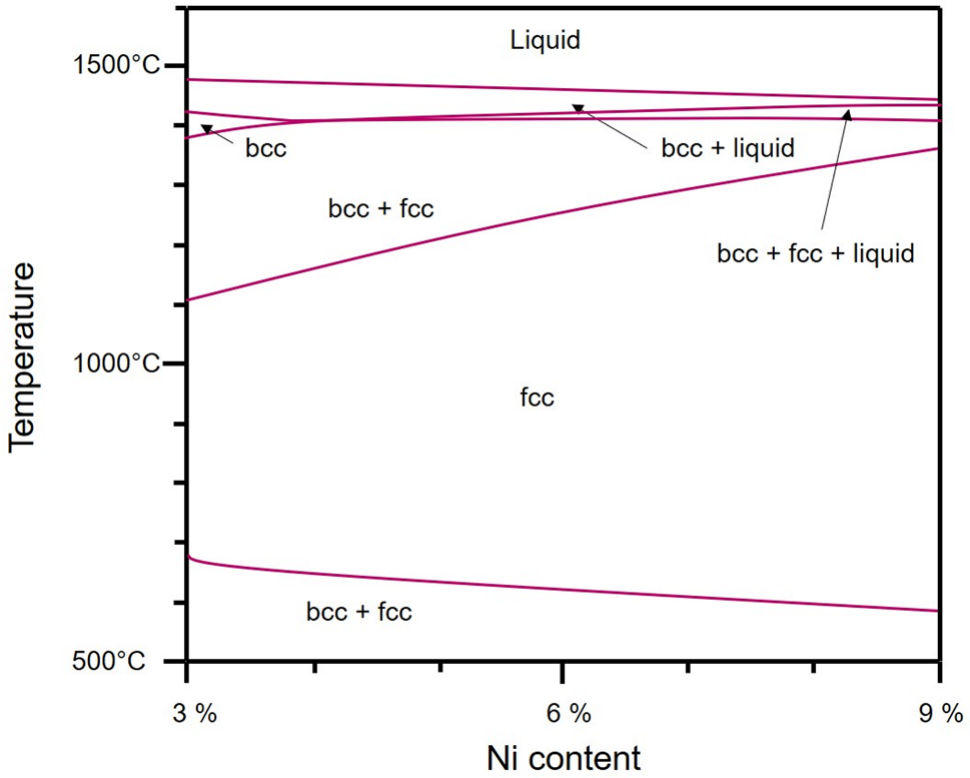
Calculated phase diagram of a CrMnNi steel with 16 wt.% Cr, 6 wt.% Mn and varying Ni contents (thermodynamic equilibrium). The carbon and nitrogen contents were set to 500 ppm and 400 ppm, respectively. The shift in phase transformation temperatures imposed by the high cooling rates being characteristic for the PBF-LB/M process is not considered. See text for details.

Depending on the chemical composition given in Table 1, the resulting grain morphologies as well as phases and their fractions are fundamentally different (cf. Fig. 1), although the same parameter settings were applied. It has to be emphasized at this point that the conditions in focus have not been processed by any thermal and/or thermomechanical post-treatment, the focus is on the PBF-LB/M as-built conditions, which have been processed at a relatively low platform temperature of 200 °C. The PBF-LB/M-16-6-9 steel exhibits a nearly fully austenitic microstructure after cooling. Similarly, in case of the PBF-LB/M-16-6-6 the austenitic microstructure is dominant, however, here the grain morphology is significantly different. Furthermore, ε-martensite (being indexed as hcp phase by EBSD) is present. Fundamentally different is the appearance of the PBF-LB/M-16-6-3 phase structure. The as-built microstructure is dominated by the bcc phase, accompanied by the presence of fcc and hcp phases with different geometric appearance, i.e., needle shaped structures. To rationalize the different microstructural evolution in the respective steel conditions, a phase diagram was calculated (cf. Fig. 5). It has to be noted that this diagram has been calculated and, thus, only is fully valid for the equilibrium state. However, as has been shown in numerous studies (e.g., in^30^), based on such equilibrium data (considering the fact that the absolute values of liquidus and solidus lines as well as the overall appearance of individual phase fields will be affected by the rapid nature of solidification and subsequent cooling) microstructure evolution imposed by the thermal history of AM processes can be estimated. The phase diagram indicates that the 3 wt.% nickel steel solidifies in a fully bcc microstructure, whereas the fcc phase is stabilized by an increased nickel content. Thus, the 6 wt.% and 9 wt.% Ni steels solidify in a dual phase microstructure at equilibrium. With decreasing temperature, the austenitic phase becomes stable in all investigated steel compositions for equilibrium conditions.

The steel with 9 wt.% Ni reveals a microstructure being characteristic for fcc materials processed by AM without any solid-state phase transformation upon solidification^50–52^. The microstructure evolution is strongly affected by the stabilization of the fcc phase with higher nickel content (Fig. 5). At a temperature below 600 °C the steel is supposed to transform into a bcc + fcc phase according to the equilibrium phase diagram. However, the partial transformation into bcc phase was not detected in present work. This can be attributed to the high heating and cooling rates and the fact that thermodynamic data available are less precise at low temperatures^42^.

For the 16-6-6 steel, mainly fcc phase was detected as expected, however, the grain morphology differs compared to the 16-6-9 alloy. As was already shown by Guenther et al.^29^, multiple phase transformations can promote grain refinement in the PBF-EB/M process. Although the process temperatures are considerably higher in the PBF-EB/M process, similar grain morphologies prevail after PBF-LB/M at a build plate temperature of 200 °C. In comparison to the 16-6-9 steel, the fcc phase field is smaller (c.f. Fig. 5), which is thought to promote multiple phase transformations in accordance to Guenther et al.^29^. Thus, although the initial solidification path is equal to the 9 wt.% Ni steel, the shift of phase transformation temperatures between bcc + fcc and fcc phase fields seems to affect the grain evolution. For bcc alloys, it was rationalized that those phase transformations generate dislocations and internal stresses, respectively, eventually leading to the formation of subgrains, which can further evolve, or serve as nucleation points for the development of new grains^24^. Furthermore, the evolution of the hcp phase takes place. Induced by a regular arrangement of stacking faults, the austenitic ABCABC stacking sequence is changed to ABAB being indexed as hexagonal ε-martensite^53,54^. As the hcp phase is not predicted by the phase diagram, since it represents a non-equilibrium transformation, stacking faults and partial dislocations, respectively, are expected to be generated due to high internal stress (details will be discussed later in this section). In contrast to 3% δ-ferrite in the cast material, no bcc phase was found in the as-built microstructure^42^. As a consequence, it is thought that only the upper half of the calculated phase diagram seems to be important to rationalize microstructure evolution for the AM material^55^.

Solidification of the 16-6-3 steel during PBF-LB/M results in a bcc dominated microstructure, however, small fractions of fcc and hcp occur. Being in line with the absent bcc + fcc transformation in the other two compositions, the complete austenitic phase transformation, which is predicted by the equilibrium phase diagram at 1100 °C, seems to be suppressed due to rapid heating, cooling and, thus, suppressed diffusion in this temperature range. Clearly, the austenitic phase is characterized by very specific morphologies in this condition. Within the ferritic melt pools acicular grains are found, whereas at the melt pool borders a more allotriomorphic austenite occurs showing austenite side plates^56^. The evolution of Widmanstatten austenite in welding is well known from literature^56–58^. It is expected that the microstructure evolves in a similar fashion here. Depending on the transformation temperature, the mechanism is either based on a diffusional nucleation and growth or a displacive formation^59^. Furthermore, the cooling rate affects the final phase fraction of austenite (value decreases at high cooling rates)^57^. The general grain morphology of the bcc phase is in line with the austenitic grains of the 16-6-9, wherefore a similar formation mechanism can be expected. The evolution of hcp phase again can be rationalized by high internal stresses between the different phases.

In order to rationalize the evolution of the hcp phase, residual stress needs to be considered. Due to high cooling rates and thermal gradients, residual stress is an important issue in PBF-LB/M^60^. In many cases, the residual stress is not only detrimental for the mechanical properties. However, stresses always promote distortion^61^. Numerous studies focus on the reduction of residual stress in PBF-LB/M by alternative scan strategies^4,62^, adapted preheating^63,64^ or specifically designed processing parameters^64^. In the present study, all specimens were manufactured with the same machine and parameter settings, such that similar thermal history and cooling conditions can be expected. However, the residual stresses in BD as well as TD are clearly different in the considered steel compositions. The 16-6-9 material shows a fully austenitic microstructure being similar to stainless steels like 316L^52,65^. This material is characterized by highest residual stress compared to the other two materials analyzed in the present work. Although the steel 16-6-6 is also mainly austenitic as well, the condition is characterized by the lowest residual stress. The reduced stress can be related to two different mechanisms, i.e., the phase transformation during processing as well as the evolution of the hcp phase. A phase transformation mostly results in a volume change and, thus, is often accompanied by constrained shrinkage or expansion. In the field of welding, it was shown that the phase transformation has a remarkable effect on residual stress^55,66–68^. The occurrence of phase transformations and the associated change of YS of the material are known to influence the final stress state^55^. In accordance to Guenther et al.^29^, the steel 16-6-6 undergoes multiple phase transformations during the AM process. The constraint shrinkage and expansion of different phases as well as the changed YS can promote the evolution of dissociated partial dislocations. The formation of ε-martensite (hcp phase) can be attributed to locally increased stress during cooling, eventually resulting in a martensitic transformation. It already has been indicated that such a mechanism may represent an effective tool to reduce residual stresses^69^. The 3 wt.% Ni steel is characterized by an intermediate stress state. Generally, the evolution of residual stress states can be rationalized in the same way as detailed for the 6 wt.% Ni steel before, i.e., by multiple phase transformations and stress-induced formation of the hcp phase. However, the differences in the prevailing microstructures have to be taken into account. In dual-phase microstructures the CTE often differs in the phases present, eventually leading to the evolution of residual stress upon cooling down to RT^48,49^. For conventionally manufactured parts, the different CTE values in fcc and bcc phases usually promote tensile stress in the ferrite and compressive stress in the austenite^48,70^. Obviously, this is not the case here. Therefore, it can be stated that several effects are superimposing each other. In direct comparison to the 6 wt.% Ni steel, the different fraction of the metastable fcc phase as well as different CTE and different YS of the bcc and fcc phases are thought to result in higher residual stress.

**Figure 6:**
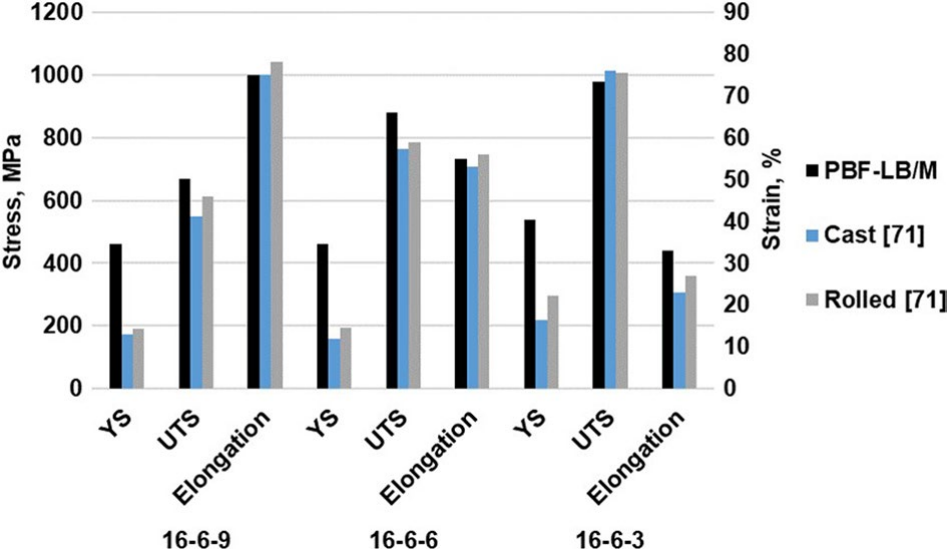
Strength and ductility of the steels with varying Ni content. Data for as-cast and rolled conditions were recompiled from^71^.

Under quasistatic loading, the materials show different deformation behaviors. In direct comparison to the as-cast and rolled conditions, the results of the AM counterparts are similar or even superior as it is shown in Fig. 6. Most importantly, YS of all AM parts is considerably higher than in case of all conventionally manufactured counterparts. In the as-cast and rolled conditions, the YS is only around 200 MPa^41,71^.

In the present study, with higher Ni content the YS of the AM parts decreased. The superior YS of the PBF-LB/M-16-6-3 material can be rationalized by the as-built dual phase microstructure. The UTS also differs highly between the materials. The 3 wt.% and 6 wt.% nickel steels reveal a pronounced hardening behavior. The different hardening behaviors again can be related to microstructure evolution. The EBSD orientation and phase maps shown in Fig. 4 depict the three different steels after deformation. The steel with 3% Ni content shows a dominant fraction of bcc phase with a minimum of retained fcc phase. The tensile behavior combined with the microstructural assessment confirms that the TRIP effect contributes to the general deformation characteristics. The characteristic plateau stage in the early stages of plastic flow, however, cannot be rationalized so far. After deformation, the 16-6-6 steel as well shows a significant change in prevailing phases. After tensile testing, the phase fractions determined are 61.2% bcc and 30.4% fcc, respectively. Since the process of phase transformation is two staged from γ-austenite, passing the intermediate ε-martensite before establishing α’-martensite^72^, a residual amount of ε-martensite (8.4%) prevails in the microstructure. Due to the high degree of deformation, the grains are decorated with small areas being characterized by the presence of different phases. Furthermore, orientation gradients within former grains promote a dynamic Hall–Petch effect^73^. The steel with 9 wt.% Ni shows no phase transformation in the examined area, however, EBSD orientation maps reveal pronounced twinning, being the reason for the high ductility in the tensile tests (TWIP). The twinning mechanism contributes mainly to the ductility and has minor effect on the strength of the material. Here, ⟨111⟩-orientated grains are characterized by extensive twinning (cf. Fig. 4c)^74,75^. The twins exhibit in general an unraveled morphology in contrast to conventionally manufactured material^76^. This is attributed to the high dislocation density within the material. The internal structures lead to a deviation from expected paths^77^.

In the present study, microstructure and mechanical properties of metastable CrMnNi steels processed by PBF-LB/M were investigated revealing a high influence of varying nickel contents. The deformation mechanisms assessed could be tailored by increasing the nickel content from TRIP to TWIP effect in accordance with changes in SFE. AM of these steels is highly attractive as it was already shown by Guenther et al.^29^. A considerably higher YS accompanied by (remaining) high ductility demonstrates the possibility to tackle the strength-ductility tradeoff also by PBF-LB/M. In addition, control of residual stress in as-built conditions is feasible. A further advantage of the process specific cooling conditions is an improved chemical homogeneity in comparison to conventionally manufactured counterparts^78^. An improved homogeneity results in superior mechanical properties, since in heterogeneous areas stress–strain localization occurs due to a locally differing SFE^79^. Besides elemental distribution, elemental evaporation needs to be taken into consideration in the field of AM. In Table 1 the chemical composition of powder and PBF-LB/M material is shown. The results reveal an evaporation of manganese, which needs to be considered during alloying. Changed process parameters may have an influence on the evaporation as shown in PBF-EB/M as well^29,30^ and need to be considered in future studies. However, in dependence of the experimentally determined elemental vaporization the steel composition could also be adapted leading to tailored mechanical properties in case of TWIP/TRIP steels since microstructure can be altered easily due to a forced evaporation^80,81^. The unique as-built dual-phase microstructure presented for the 16-6-3 steel in combination with a tailored chemical homogeneity exploiting the process inherent high cooling rates of PBF-LB/M represents a promising pathway toward enhanced mechanical strength. Although the material is less ductile in direct comparison to the other compositions, the ductility of the material is still sufficient with more than 30% elongation at fracture. However, several research gaps are still prevailing. These need to be addressed in future publications focusing on elementary deformation mechanisms and their interaction to substantiate their impact on the mechanical properties in the AM materials.

## Conclusions

In the present study, three different CrMnNi steels with varying nickel content processed by PBF-LB/M were examined. Via EBSD, the differences in microstructural evolution imposed by the process related thermal history were investigated. Only as-built conditions were characterized. Residual stress states as well as mechanical properties were assessed. Considering tensile testing, different deformation mechanisms were found to be active. The following conclusions can be drawn from the results presented:

1. The chemical composition highly influences the solidification behavior of CrMnNi steels. With a nickel content of 3 wt.% the material solidifies in the bcc phase; upon cooling, fractions of fcc and hcp phase are present. An increase of nickel leads to a higher stability of the fcc phase. Thus, the steel with 6 wt.% nickel shows a fcc dominated microstructure with a minor fraction of hcp phase. For the highest nickel content (9 wt.%) the microstructure is fully austenitic.
2. In dependence of the nickel content, fundamentally different solidification microstructures were found in the PBF-LB/M specimens. These differences can be attributed to different sequences of phase transformations during processing. Multiple phase transformations promote the evolution of a fine-grained microstructure and even can result in different grain morphologies of individual phases. The evolution of ε-martensite (hcp phase) is thought to be triggered by high local stress in the fcc austenite.
3. The residual stress states are different for each steel. Decrease of residual stress can be attributed to two different elementary mechanisms: numbers of phase transformations in a characteristic high-temperature region and stress-induced martensitic transformation. Based on an improved steel design supported by future in-operando measurements, residual stress states seem to be tailorable.
4. The tensile tests reveal different mechanical properties in dependence of the nickel content. With an increasing nickel content, YS and UTS decrease, at the same time the ductility is increased. The UTS and elongation at fracture are competitive to conventionally manufactured counterparts, whereas the YS is significantly increased. The varying nickel content leads to a changed SFE resulting in different active deformation mechanisms. The steels with the lowest and medium nickel content reveal a TRIP effect under deformation. At 9 wt.% of nickel the TWIP effect is dominant.

## Materials and methods

In the present study, cuboids with a size of (l × w × h) 10 × 10 × 40 mm^3^ were manufactured via PBF-LB/M using a SLM280^HL^ by SLM Solutions (Germany) considering three different pre-alloyed powder grades with a variation in nickel content. In accordance with the targeted composition of 16 wt.% Cr, 6 wt.% Mn and 3, 6 or 9 wt.% Ni, the specimens are referred to as 16-6-3, 16-6-6 and 16-6-9, respectively. The initial powders were gas atomized and supplied by TLS Technik GmbH (Germany) with a particle size ranging between 40 and 150 µm. In the present study, only powder being characterized by this relatively large particle size was available. It was expected that the steels being in focus can be processed on this high level of efficiency due to their balanced characteristics (being the scope of present work). Preliminary process optimization allowed to manufacture parts of a high relative density (cf. Supplementary data, Fig. S1). A more generalized validation of coarse powder (including other alloy systems, e.g., pure iron) for PBF-LB/M process needs to be considered in future work, especially in light of particle distributions after multiple reuses of powder. For the PBF-LB/M process only the particle fraction below 100 µm was used, i.e., the powders were sieved. The PBF-LB/M system was operated with a laser power of 237.5 W and scanning speed of 700 mm/s for all materials. The hatch distance was 0.1 mm and the layer thickness 0.05 mm. The scan pattern was meandering with a rotation of 90° in every consecutive layer. The build plate was heated to 200 °C.

Tensile specimens with a gauge section of 8 × 3 × 1.6 mm^3^ were wire cut via electro-discharge machining (EDM). The surfaces of the specimens were ground to P1200 to eliminate residue stemming from EDM. Tensile tests were conducted parallel to BD using an MTS Criterion Model 43 in displacement control at a crosshead speed of 2 mm/min (corresponding to a nominal strain rate of 0.004 1/s). The strain was measured up to 30% using an extensometer directly attached to the surface. For all strain values above, the strain was recalculated from displacement data. For each condition, three tensile tests were conducted.

The chemical composition of the powder as well as the PBF-LB/M bulk material was determined by X-ray fluorescence spectroscopy, inductively-coupled plasma spectroscopy, carrier gas hot extraction for nitrogen and combustion gas analysis for carbon. For the investigation of phases and crystallographic orientation, a scanning electron microscope Zeiss ULTRA GEMINI equipped with an electron backscattered diffraction (EBSD) detector was used at an acceleration voltage of 20 kV. For EBSD, the surfaces were further ground to P4000 and finished by vibration polishing with a colloidal silica suspension (MasterMet 2) for 16 h. Grain sizes were evaluated by Bruker Software (based on a misorientation angle above 15°).

Residual stresses were measured at the side surface of the bulk specimens for each chemical composition. The point of measurement was located at the as-built surface at a built height of 20 mm. Residual stresses were evaluated for the γ phase for all three chemical compositions. The measurements were conducted using high energy-dispersive X-ray diffraction (ED-XRD) with an energy range of 20-100 keV at the P61A beamline of “Deutsches Elektronen-Synchrotron”, DESY. The beam size was 0.5 × 0.5 mm^2^ covering a depth of 274 µm.

Finally, to support the microstructural discussions, the pseudo-binary equilibrium phase diagram (isoplethal section) for the alloy system Fe–16Cr–6Mn–0.05C–0.04 N– (3–9)Ni (concentrations in wt.%) was calculated using Thermo-Calc with TCFE9 database. Calculations were carried out under a pressure of 1 bar. All phases predicted by Thermo-Calc were permitted during the calculations.

## Supplementary Information

Supplementary Information.

## Data Availability

The datasets used and analyzed during the current study are available from the corresponding author on reasonable request.
