# Supplementary material for: Metastable CrMnNi steels processed by laser powder bed fusion: experimental assessment of elementary mechanisms contributing to microstructure, properties and residual stress
Source: Sci Rep. 2022 Dec 18;12:21862. doi: 10.1038/s41598-022-26052-x (PMC9760645; doi:10.1038/s41598-022-26052-x)
Supplement: Supplementary file 1 — Supplementary Information. [file 41598_2022_26052_MOESM1_ESM.docx]

**Supplementary Data for:**

**Metastable CrMnNi steels processed by laser powder bed fusion - Experimental assessment of elementary mechanisms contributing to microstructure, properties and residual stress**

*J. Richter^1,^ *, G. Bartzsch^2^, S. Scherbring^3^, A. Bolender^1^, M. Vollmer^1^, J. Mola^3^, O. Volkova^2^, T. Niendorf^1^*

*^1^* *University of Kassel, Institute of Materials Engineering – Metallic Materials, Moenchebergstrasse 3, 34125, Kassel, Germany*

*^2^ TU Bergakademie Freiberg, Institute of Iron and Steel Technology, Leipziger Strasse 34, 09599 Freiberg/Saxony, Germany*

*^3^ Osnabrueck University of Applied Sciences, Faculty of Engineering and Computer Sciences, Materials Design and Structural Integrity Laboratory, Albrechtstrasse 30, 49076 Osnabrueck, Germany*

**Corresponding Author: julia.richter@uni-kassel.de*

This Supplementary Data contains five supplementary figures (Figures S1-S5).

**
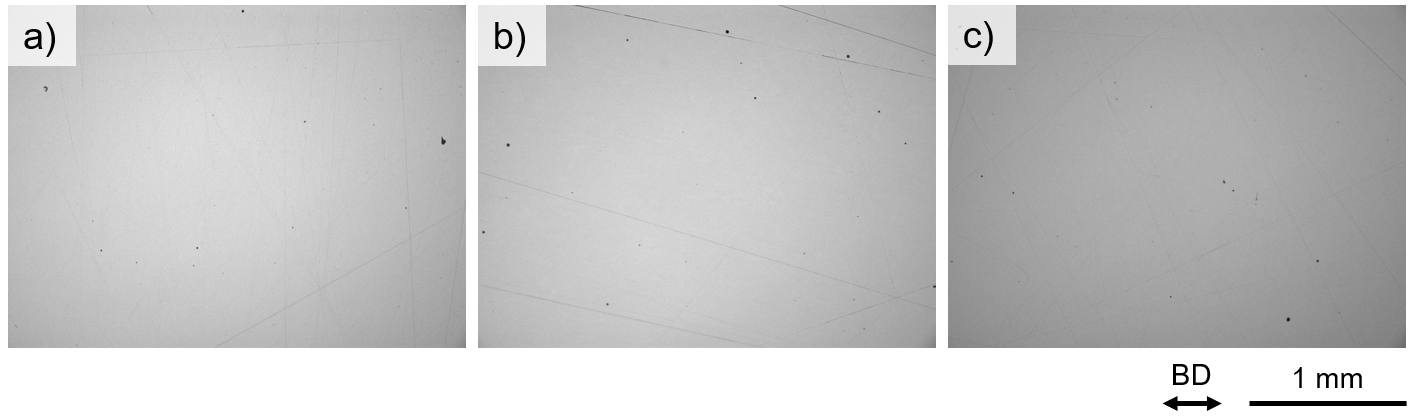
**

S1 Representative micrographs of the polished specimens revealing the densification behavior of the 16-6-X material with X = 3 % Ni (a), X = 6 % Ni (b) and X = 9 % Ni (c)


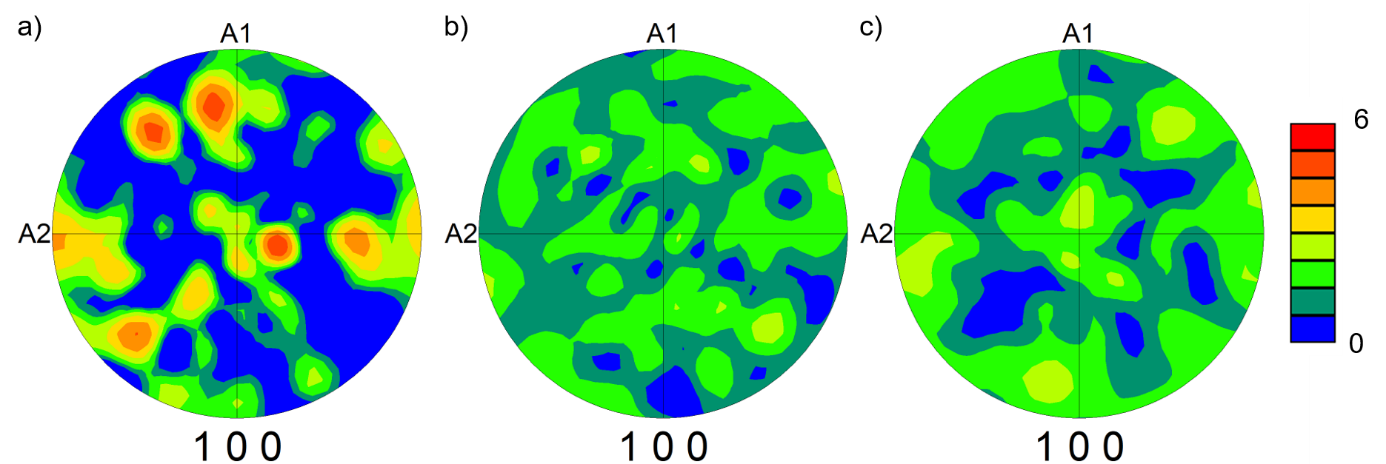


S2 The texture intensity plots of the specimens built from 9 % Ni, 6 % Ni and 3 % Ni powders are displayed in (a), (b) and (c), respectively. Texture intensities being representative for all pole figures are given to the right.


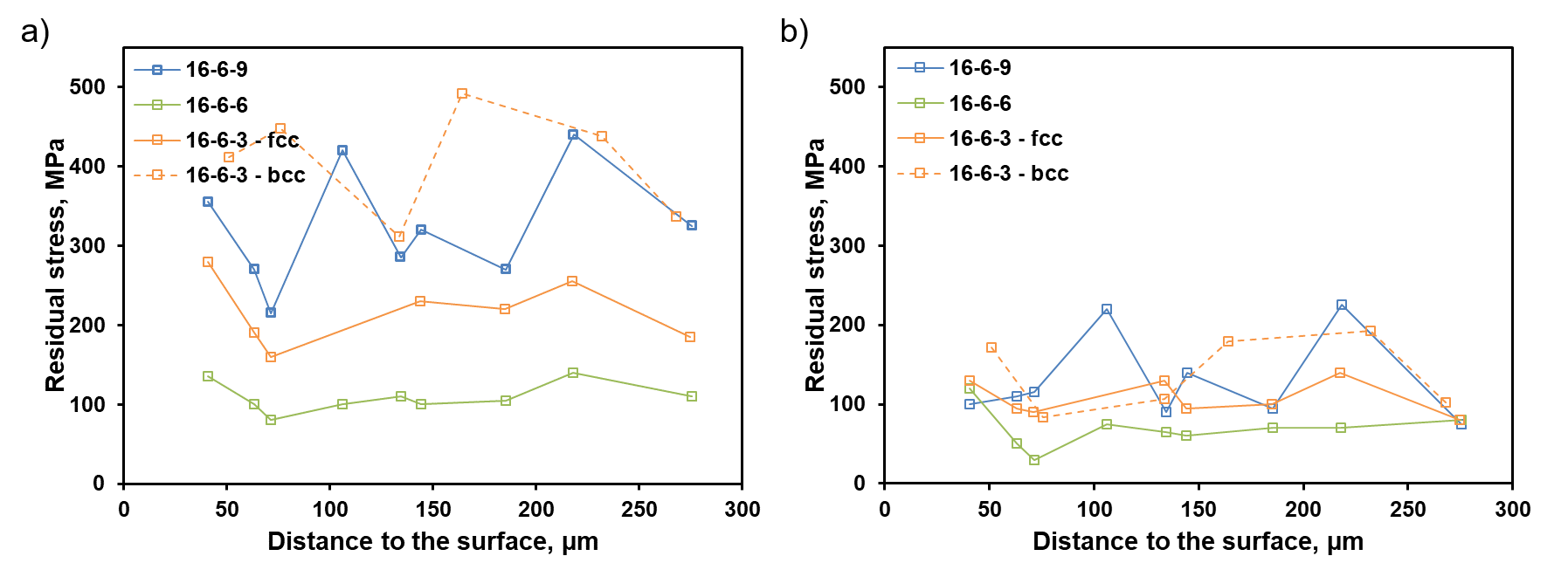


S3 Near surface residual stress distribution determined by ED-XRD analysis for PBF-LB/M material of all three steels with respect to BD as a function of distance to the surface (a) as well as in TD (b). For the 16-6-9 and 16-6-6 alloy, the stresses of the fcc phase were analyzed, in case of the 16-6-3 alloy the bcc and fcc phases were evaluated.


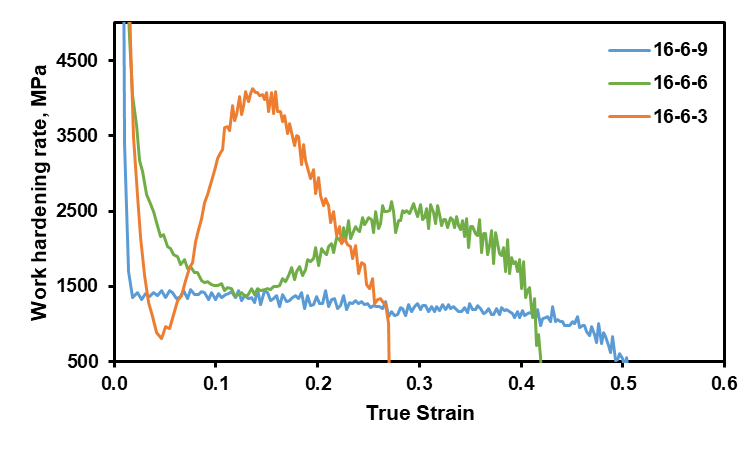


S4 True work hardening rate calculated from the true stress – true strain values; The 16-6-9 steel exhibits an almost constant work hardening rate until necking. The work hardening rate changes with the applied strain.


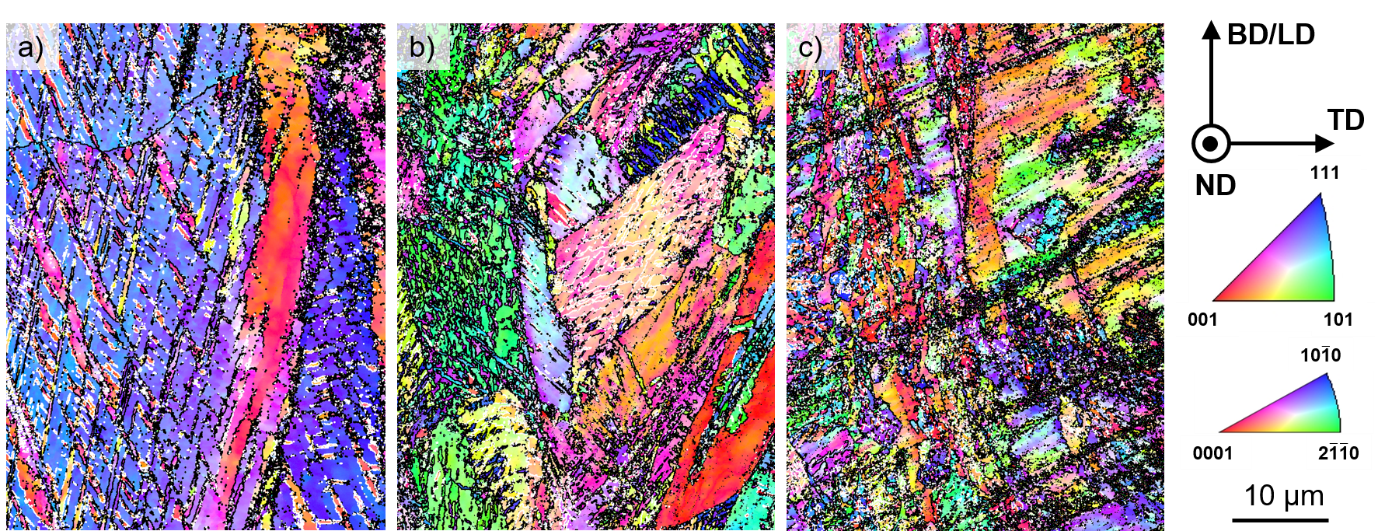


S5 EBSD orientation maps of PBF-LB/M-16-6-9 (a), PBF-LB/M-16-6-6 (b), PBF-LB/M-16-6-3 (c) after tensile testing plotted with respect to BD/LD. Grain boundary angles are highlighted in black between 15° and 60°. Twin boundaries are highlighted in white. Non-indexed areas revealed by black color are due to high local deformation. The step size for all maps was 0.075 µm.
